# Automated soccer head impact exposure tracking using video and deep learning

**DOI:** 10.1038/s41598-022-13220-2

**Published:** 2022-06-03

**Authors:** Ahmad Rezaei, Lyndia C. Wu

**Affiliations:** Department of Mechanical Engineering, University of British Columbia, Vancouver, V6T 1Z4 Canada

**Keywords:** Biomedical engineering, Brain injuries, Computer science

## Abstract

Head impacts are highly prevalent in sports and there is a pressing need to investigate the potential link between head impact exposure and brain injury risk. Wearable impact sensors and manual video analysis have been utilized to collect impact exposure data. However, wearable sensors suffer from high deployment cost and limited accuracy, while manual video analysis is a long and resource-intensive task. Here we develop and apply DeepImpact, a computer vision algorithm to automatically detect soccer headers using soccer game videos. Our data-driven pipeline uses two deep learning networks including an object detection algorithm and temporal shift module to extract visual and temporal features of video segments and classify the segments as header or nonheader events. The networks were trained and validated using a large-scale professional-level soccer video dataset, with labeled ground truth header events. The algorithm achieved 95.3% sensitivity and 96.0% precision in cross-validation, and 92.9% sensitivity and 21.1% precision in an independent test that included videos of five professional soccer games. Video segments identified as headers in the test data set correspond to 3.5 min of total film time, which can be reviewed through additional manual video verification to eliminate false positives. DeepImpact streamlines the process of manual video analysis and can help to collect large-scale soccer head impact exposure datasets for brain injury research. The fully video-based solution is a low-cost alternative for head impact exposure monitoring and may also be expanded to other sports in future work.

## Introduction

Repetitive sports head impact exposure may not only increase the risk for sustaining acute brain injury such as concussion^1,2^, but also lead to cumulative brain changes in the long term^3^. However, there are limited data to quantify the correlation between impact exposure and brain health consequences. In soccer, head impacts are common since players often use their head to redirect the ball. In fact, soccer heading represent approximately 90% of head impacts in soccer, and the remaining impacts are mostly unintentional player-to-player or head-to-ground impacts^4^. It is unclear whether intentional soccer heading, which are more frequent but mostly lower-severity than unintentional head impacts, may have deleterious effects on the brain. Exposure to a single session of controlled heading has been associated with increases in reported concussion symptoms^5^, alterations in postural control^6^, and increases in corticomotor inhibition with impaired memory function^7^. For players that self-reported long-term exposure of 885 to 1800 headers per year, brain white matter microstructure abnormality and neurocognitive dysfunction has been found^8^. However, some studies have not found significant neurocognitive performance or neuropsychological changes associated with short-term soccer heading exposure^9,10^. Using self-reported impact estimates in some studies can be a potential reason for the mixed results in the literature. Such subjective exposure reports may not provide accurate estimates of head impact count^11^. Because of the inconclusive results of repeated soccer head impact exposure, longitudinal research to correlate accurate exposure measurements with brain outcomes is recommended^12^. Therefore, collection of accurate large-scale head impact exposure data is a vital step to study brain injury mechanisms.

Some studies have used wearable head impact sensors to objectively and quantitatively monitor impact exposure^13^. These sensors contain inertial measurement units (IMUs) to measure head kinematics, and typically set a linear acceleration threshold (e.g. 10g) to detect and record impact events^14–16^. Despite the simplicity of this method, it has limited accuracy in head impact detection as it fails to record any information about the events occurring below the threshold. Therefore, selecting the right threshold is important but it is also highly subjective, which can introduce bias in the exposure data^17–19^. To accurately quantify head impact exposure, methods that can detect most impacts (high sensitivity) without a substantial number of false positive detections (high precision) are required. Applying a low acceleration threshold may be a strategy to increase impact detection sensitivity at a cost of high false positive rate, while increasing the threshold to improve precision can lead to increased false negative rate^19,20^. Furthermore, limited kinematic accuracy in sensors leads to uncertainty in the estimated head accelerations for impact triggering^21,22^. Some sensors have combined the simple acceleration threshold method with additional filtering algorithms to remove false positive recordings. However, laboratory and field evaluation studies have shown poor performance of such algorithms^21,23–25^. Table 1 gives a summary of sensor-based head impact studies in soccer. A few studies have quantified impact detection performance of the applied sensors and found limited sensitivity and precision. Moreover, wearable head impact sensors are costly for large-scale deployment and exposure tracking, and they may not always be readily accepted or regularly worn by sports participants.

**Table 1:** Previous head impact exposure studies in soccer.

Study	Players and Games	Sensor	Detection method	Verification method	Sensitiviy	Precision
Hanlon et al.^26^	23 players	HIT (headband)	10g impact threshold	Video confirmation	–	–
McCuen et al.^27^	53 players, 1 season	XPatch (skin patch)	20g impact threshold	No confirmation	–	–
Caccese et al.^16^	25 players, 14 games	SIM-G (headband)	10g impact threshold	Video confirmation	–	–
Lynall et al.^28^	22 players, 57 sessions	XPatch (skin patch)	10g threshold with proprietary algorithm	No confirmation	–	–
Press et al.^4^	26 players, 46 sessions	XPatch (skin patch)	10g threshold with proprietary algorithm	Video confirmation	85.9	16.3
Lamond et al.^29^	23 players	SIM-G (headband)	10g impact threshold	Visual confirmation	91.4	6.1
Chrisman et al.^15^	46 players	XPatch (skin patch)	15g impact threshold	Visual confirmation	–	–
Rich et al.^30^	4 players, 14 sessions	Mouthpiece	5g impact threshold	Independent video analysis	69.2	80.3
Patton et al.^31^	72 players, 41 sessions	SIM-G (headband)	16g threshold with proprietary algorithm	Video confirmation	–	69.5
Miller et al.^14^	7 players, 31 sessions	Mouthpiece	10g impact threshold	Independent video analysis	50.6	–
Tomblin et al.^32^	14 players, 66 sessions	Mouthpiece	5g impact threshold	Independent video analysis	43.1	–
Filben et al.^33^	14 players, 96 sessions	Mouthpiece	5g impact threshold	Video confirmation	–	20.0

Considering the limitations of impact sensors, video analysis is recommended to improve the accuracy of impact exposure data^34^. Most studies use video data only to confirm recordings of the sensor^4,16,26,31^, which is limited since this approach does not identify potential false negatives and may underestimate impact exposure. Therefore, other studies have conducted independent analysis of the video to identify all potential exposure events based on human reviewer observation^14,20,35^. Video information has also been used to extract contextual factors characterizing head impacts, such as impact location, impacting object and impact type^14,16,20^, which cannot be fully informed by IMU-based impact sensing. However, video analysis is a time-consuming process that requires substantial human resources. In a previous study, fourteen trained raters reviewed 163 hours of video to fully verify 217 head impacts^20^. Generating large-scale head impact exposure data using independent video analysis would be a cost-prohibitive and lengthy task.

Automatic analysis of sports games using video information has been extensively studied in computer vision research^36^. Computer vision algorithms, more specifically deep learning (DL) techniques, have been applied to automate tasks such as player/ball detection and tracking, player pose estimation, game reconstruction, and generation of game statistics^36^. In this work, the feasibility of a video-based approach for automatic soccer header detection through DL algorithms is investigated. The goal of this study is to develop a more efficient and less resource-intensive approach for video-based head impact exposure estimation.

**Figure 1:**
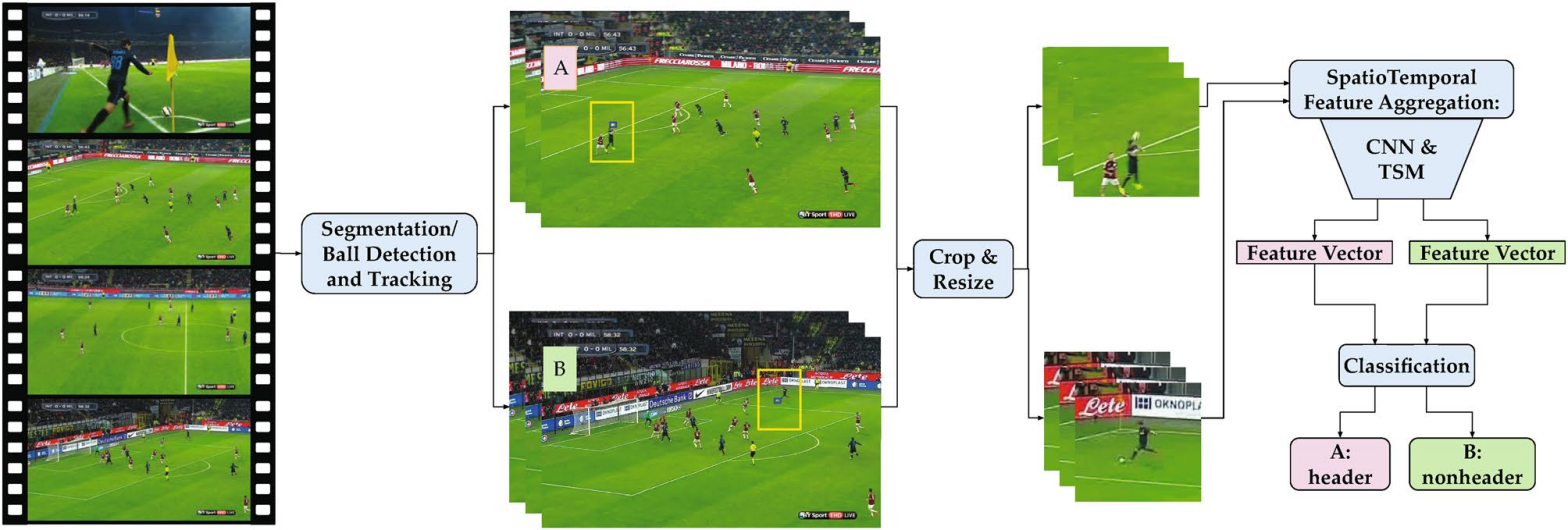
Overview of the proposed header detection algorithm. The video is divided into short segments and ball position is detected and tracked in each segment. Each frame is then cropped around the ball position. Spatiotemporal features of each cropped video segment are extracted using a convolutional neural network (CNN) and temporal shift module (TSM). The extracted feature vector is then classified as a header or nonheader event in the last step of the algorithm.

## Methodology

DeepImpact, a DL algorithm with the novel application of automatic header impact exposure tracking using a single-view broadcast soccer video is proposed. This data-driven algorithm uses video information and computer vision algorithms to detect headers, which are defined as any contact between a player’s head and the soccer ball. Header detection in the video can be categorized as a video understanding problem in computer vision research, which consists of the identification and temporal localization of actions in a video.

### Algorithm overview

The video data has two types of information: visual information present in each frame of the video as well as dynamic temporal information that can be obtained from consecutive frames. DL-based research in video understanding follows a general structure to use this spatiotemporal information. The common method comprises of extracting visual features in the spatial domain from each frame using convolutional neural networks (CNN), then temporally aggregating the features of consecutive frames to generate a video descriptor^37–39^. The descriptor is then used to train an action classifier. This is a complete data-driven approach in which the algorithm is trained on a dataset comprised of video samples from each action.

Figure 1 shows the proposed framework, which consists of 5 steps. The input video is divided into short segments and in each segment, ball position is detected using a DL object detector algorithm. A ball tracking algorithm is then applied to the detected ball positions to improve the estimation of ball location. Using the estimated ball position, each frame is cropped around the location of the ball. Next, spatial and temporal information of all frames in each video segment are extracted and aggregated into a feature vector. Similar to the ball detection network, a DL-based spatiotemporal feature aggregation network is used, which is trained using a soccer header dataset. Lastly, a neural network classifier is trained to classify the visual-temporal features as a header or nonheader event. The purpose of the ball detection and frame cropping step is to minimize distractions in visual information. Soccer headers only happen in the presence of a soccer ball. Considering the relatively small size of the ball and players in broadcast soccer video, cropping the frames helps to preserve an area of the image relevant to header events.

### Video segmentation

Frames of the input video were extracted and segmented based on the selected segment length, which is the number of frames in each segment. Segment lengths between 5 to 15 frames were selected and the performance of the algorithm was evaluated for each length using cross validation on the full dataset (see “Performance evaluation”). A segment length of 11 frames yielded the best results. For header segments, the frame that contained the header moment was centred.

### Ball detection

The ball detection algorithm used in this work is a modification of the DL-based object detection algorithm, You Only Look Once v5 (YOLOv5)^40^. YOLOv5 is currently one of the best performing object detection algorithms. A series of convolutional layers are applied to the input image to extract its visual features for object identification inside the image^40^. The algorithm detects each object by estimating a bounding box around it as well as a probability that represents its confidence in the detected category of the bounding box. YOLOv5 has 4 model sizes, YOLOv5s/m/l/x, which have different numbers of learnable network parameters. A larger model with more learnable parameters can achieve higher accuracy considering that the training dataset is large enough to avoid over-fitting. However, more parameters require higher graphics processing unit (GPU) memory in the training process, which limits the possible highest batch size. In this study, YOLOv5l was used for the ball detector network because it was the largest size that allowed a minimum batch size of 32 on an NVIDIA RTX 3080 Ti GPU.

#### Soccer ball dataset

SoccerDB^41^ dataset was used to train the ball detection network. SoccerDB is a large-scale soccer video understanding dataset, which contains videos of 53 full-match and 18 half-match professional male soccer games. It has annotations for object detection, including 64,232 images with ground truth (GT) bounding box of the soccer ball. Furthermore, SoccerNet-v2^42^ dataset was used to achieve a larger dataset size as well as higher variability in the images of the training dataset. SoccerNet-v2 is a soccer activity recognition dataset that contains high quality videos of 500 professional male soccer games. Ball positions in 55,000 randomly selected images that were extracted from the SoccerNet-v2 were labeled. Overall, the soccer ball dataset contained ground truth ball position in 119,232 images from 221 soccer games. These images contained a diverse set of scenes including different stadiums, lighting conditions, camera positions/angles, ball size/color/location, and scenes with a partially covered ball. This diversity helped to train a robust ball detection network.

#### Training

The ball detection network was first pre-trained on the Common Objects in COntext (COCO) dataset^43^. COCO is a large-scale object detection dataset that contains 330,000 images with 80 object categories^43^. Weights of the pre-trained network were used to initialize the ball detector network. The pre-training helps to achieve a more accurate ball detection network and reduce the necessary training time^44^. The last layer of the network was modified for two object categories (ball and background). The network was then trained on the soccer ball dataset.

The suggested training hyper-parameters of YOLOv5 were used in the training process. Initial learning rate was set to 0.001 and the Adam optimizer was used in all experiments. Batch size was set to 32 and the network was trained for 300 epochs. The soccer ball dataset was randomly split into 100,000 images for training and 19,232 images for validation. Average precision (AP) was selected as the validation metric, which is commonly used to evaluate the performance of object detection algorithms^40,45^. Performance of the network was evaluated after each epoch and the best performing set of weights with the highest AP were used for the next steps of the header detection process. The network provides a confidence score for its detections. F1-score^46^, which combines sensitivity and precision into one metric was calculated to determine the best threshold on this confidence. Detections with a confidence lower than the selected threshold were considered as background.

### Ball tracking

Some frames could contain false positive ball detections or fail to detect the ball. To reduce the effect of such noisy detections, a Kalman filter (KF) based ball tracking method was implemented. KF has been extensively used for visual object tracking^47,48^ and the same approach was followed in this work. A constant speed model was used to model ball movement, with the state vector defined as

1 $$\begin{aligned} \mathbf {x_k}=\left[ u,v,\dot{u},\dot{v}\right] ^T, \end{aligned}$$

where *u* and *v* denote the horizontal and vertical coordinates of the center of the bounding box, respectively, with time derivatives $\dot{u}$ and $\dot{v}$. Detailed formulation of the KF can be found in Forsyth and Ponce^49^. In this application, the ball detection network provided the bounding box location in each frame as the measurement of the KF and the constant speed model provided the prediction of the ball location. The parameters of the measurement covariance matrix were set to 15 pixels for horizontal and vertical coordinates to optimize performance.

To initialize *u* and *v*, the detected ball with the highest confidence score in the first frame of the video segment was used, with $\dot{u}$ and $\dot{v}$ initialized to zero. Process noise covariance matrix was initialized with 20 pixels for location parameters and 5 pixels/s for speed parameters.

#### Outlier rejection

An outlier rejection method was applied to the ball detections in each step of the filter to maintain its stability. Ball location should not vary considerably between consecutive frames. Therefore, the horizontal and vertical distance between the detected ball location using the ball detection network ($u_d$ and $v_d$) and the predicted ball location using the constant speed model ($u_p$ and $v_p$) were calculated in each frame. If either the *u* or *v* distance was higher than a threshold value *d*, the KF innovation ($[u_d-u_p, v_d-v_p]$) value was set to zero and no correction was applied to the predicted location of the ball. For optimized performance, *d* was set to five times the width of the bounding box in the first frame of the video segment. Similarly, if the ball detection network did not detect any ball in a frame and no measurement was available for the filter, the innovation value was set to zero. Furthermore, KF was only applied to video segments that had at least five frames with a detected ball.

### Frame cropping

Using the estimated location of the bounding box, each frame was then cropped to center the bounding box. The cropped image size was selected to be 10 times the size of the bounding box. In the next step of the algorithm, temporal feature aggregation network needs all frames of the input video to have the same size. Therefore, cropped images were resized to a fixed size (640,480) using the bicubic interpolation technique, which employs polynomial-based interpolation to enlarge the image^50^.

### Spatiotemporal feature aggregation

A deep learning algorithm called temporal shift module^51^ (TSM) was used for spatiotemporal feature aggregation. TSM is an efficient method used for video understanding, which compares well with other potential approaches for temporal modeling of video such as 3D CNNs^52^, recurrent neural networks^38^, and attention mechanisms^53^. TSM employs 2D CNNs to extract visual features of each input image and fuses the extracted features using a temporal shift mechanism^51^. Using this algorithm, spatiotemporal features of each video segment can be learned using one neural network in an efficient manner. Because TSM employs 2D CNNs, common deep CNN structures such as VGG^54^ and ResNet^55^ could be used for visual feature extraction. ResNet-50 structure, which is 50 layers deep was used for visual feature extraction. Using deeper ResNet-101 structure did not make any improvement on the results.

The size of the input tensor to the feature aggregation network was (11, 3, 640, 480), in which 11 is the number of frames, 3 is the number of color channels in each frame, and (640, 480) is the resolution of the image. The network maps this input to a $1\times 2048$ feature vector. For final classification of this feature vector as a header or nonheader event, two fully-connected neural network layers were added to the output of the feature aggregation network to generate a $1\times 2$ probability vector. Parameters of the feature aggregation network and the classification layers were trained using a soccer header dataset.

#### Dataset

For training the feature aggregation network, a dataset containing both header and nonheader events was generated using broadcast soccer video. Three source datasets were used to obtain these events: SoccerNet-v2, SoccerDB, and publicly available soccer videos from the internet. Table 2 shows the details of this dataset. In total, 4843 header events from 51 games and 33,034 nonheader events from 185 games were included in the dataset. Events were selected from videos with 25, 30, and 50 frames per second. For header events, three trained video raters reviewed the game videos to identify any possible head contact with the ball. One of the authors then assessed all the identified contact events to ensure only headers are included in the dataset. For nonheader events, equal numbers of video segments were randomly selected from each game. One of the authors reviewed all nonheaders to ensure the randomly selected segments did not contain any headers. The labeled header and nonheader event dataset was then used to train and validate the algorithm.

**Table 2:** Number and source of events in header dataset.

Event	Source	Number of games	Number of events	Events with GT ball
Header	SoccerDB	20	1850	1850
	SoccerNet-v2	26	2741	2741
	Internet	5	252	252
	Total	51	4843	4843
Nonheader	SoccerNet-v2	185	33,034	2904

#### Training

Similar to the ball detection network, a network pre-trained on the Kinetics^56^ dataset, which is a general human action recognition dataset with 306,245 short video clips of 400 action classes was used. Parameters of the pre-trained network were used as the initialization point of the feature aggregation network and training was continued on the soccer header dataset. For training, the learning rate was set to 0.001 using a stochastic gradient descent optimizer. 0.0005 was used as the weight decay rate of the optimizer. The network was trained for 30 epochs with a batch size of 32. For the hyper-parameters of the TSM mechanism, the same settings as the original TSM work was used^51^. Performance of the algorithm was assessed on the validation dataset after each epoch using the evaluation metrics and the network weights that yielded the best metrics result were selected.

### Performance evaluation

For evaluating the performance of DeepImpact algorithm in differentiating the header events from nonheaders, its detections were compared with the ground truth label of each video. Performance metrics of sensitivity, precision, specificity, and accuracy were calculated using the number of true positives (TP), false positives (FP), true negatives (TN), and false negatives (FN). Sensitivity and precision are especially important for assessing the performance of the header detection algorithm. Sensitivity shows classifier’s performance in identifying all header events of the video while precision reflects the number of nonheader events that are classified as headers (FP). These metrics are defined below.

2 $$\begin{aligned} \textit{sensitivity}&= \frac{TP}{TP+FN}, \end{aligned}$$

3 $$\begin{aligned} \textit{precision}&= \frac{TP}{TP+FP}, \end{aligned}$$

4 $$\begin{aligned} \textit{specificity}&= \frac{TN}{TN+FP}, \end{aligned}$$

5 $$\begin{aligned} \textit{accuracy} =&\frac{TP+TN}{TP+FP+TN+FN}. \end{aligned}$$

The performance of the header detection network was evaluated by conducting 5-fold cross validation. The header dataset consists of a diverse set of different soccer games. Cross validation can reduce potential bias in assessment of the performance. The unshuffled dataset was split to 5 partitions and each partition was used as the validation set in one pass of training. The averaged metrics were used for assessment.

#### Performance evaluation using test dataset

To further evaluate the performance of the header detection algorithm, this network was tested on 5 unseen independent soccer games from the SoccerNet-v2 dataset. The entire duration of the test games were processed into header and nonheader event segments, which is different from the training/validation dataset where a selected number of random nonheader events were used from each game. Using this analysis, the performance of the header detection algorithm in its final application scenario, where headers should be detected in full soccer videos was assessed.

#### Effect of algorithm design

To evaluate the contribution of each element of the proposed header detection algorithm on its overall performance, the network was trained and validated in different settings, as described below.

**Setting 1 - Classification of Single Uncropped Frame:** In the simplest setting, video segmentation, ball detection, frame cropping, and temporal feature aggregation steps were removed. An image classification network (ResNet-50) was trained to classify each frame of the video as a header or nonheader event. No temporal information was used in this setting and full-size frames were used without cropping them around the ball position.

**Setting 2 - Classification of Uncropped Video Segments:** In this setting, ball detection and frame cropping steps were removed from the pipeline. Video segments consisting of multiple full-size frames were used to train the TSM network. While temporal information was used in this setting, potentially irrelevant and distracting spatial information were included as frames were not cropped using ball detection.

**Setting 3 - Full Algorithm:** All elements of the algorithm were included in this setting. Other two settings were compared with this setting to determine the relative contributions of temporal information and frame cropping on header detection performance.

## Results

### Ball detection

**Figure 2:**
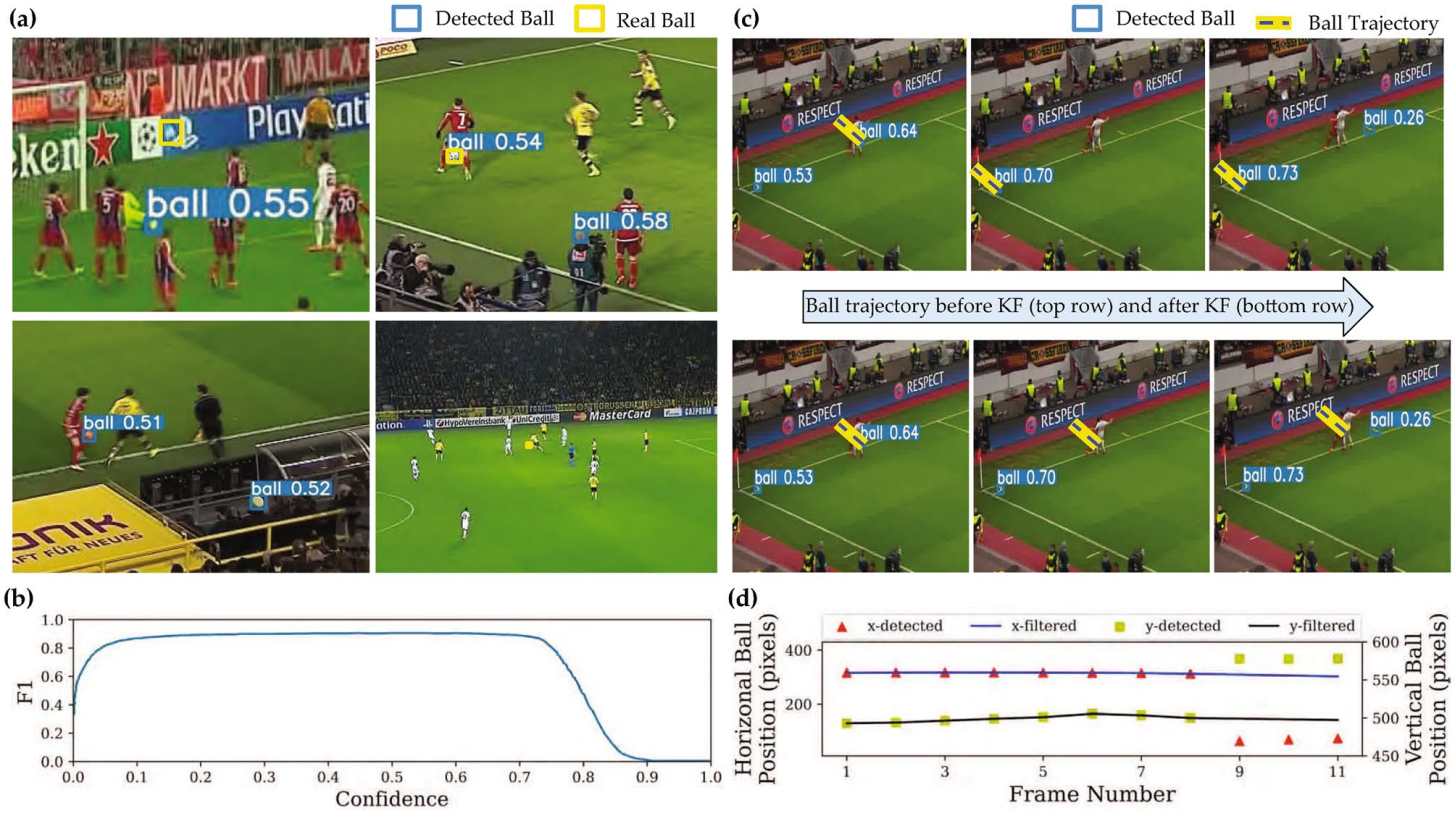
Performance of the ball detection network and ball tracking. (**a**) Ball detection failed in some cases when the ball was occluded by a player, moving on a non-green background, or had very small size (only few pixels). (**b**) F1 score-confidence curve shows the balance between the sensitivity and precision of ball detection when the confidence threshold is varied. (**c**) In frames 8–10 of the segment, the ball is occluded by the players in frames 9 and 10 and remains undetected while other outliers are present in each frame. KF rejects the outliers and uses its prediction to correctly estimate the location of the ball. (**d**) Horizontal and vertical coordinates of the detected and filtered ball location are shown. KF estimates a smooth track for the ball movement by rejecting the outliers.

The ball detection network had 0.90 AP using an intersection over union^45^ (IoU) of 0.5 ($AP^{IoU=0.50}$). Using IoU values in a range of 0.50 to 0.95 with 0.05 step size, mean AP was 0.40 ($AP^{IoU=0.50:0.05:0.95}$). In general, successful detection of the ball proved to be a challenging task. The common cases of incorrect detection included (Fig. 2a): 1) ball was fully or partially occluded by a player; 2) other objects in the background such as a body part were detected as a ball; 3) ball size was extremely small.

The ball detection network provided a confidence score between 0 and 1 for its detections. We selected a threshold value of 0.15 on this confidence, which provided an F1-score above 0.8, showing good balance between sensitivity and precision of ball detection (Fig. 2b). Detections with confidence above the threshold were used in ball tracking, while any detection with lower confidence was ignored.

### Ball tracking

To demonstrate the effectiveness of ball tracking, Fig. 2c shows a video segment where the ball was occluded in the last three frames. Before applying KF, an outlier object was incorrectly detected as the ball (Fig. 2c). It can be seen that after KF was applied, outlier detections were rejected and KF yielded a correct smooth track for the ball, even though it was occluded by the players (Fig. 2d). Outlier rejection happened in 47% of the video segments of the training and validation dataset.

**Table 3:** Header detection performance on the validation dataset.

Metric	Sensitivity	Precision	Specificity	Accuracy
Cross validation	95.3	96.0	99.4	98.9

**Table 4:** Header detection performance on the test games.

	Game 1	Game 2	Game 3	Game 4	Game 5	Total	Average
TP	78	132	90	127	80	507	101
FN	4	11	6	10	8	39	8
FP	392	485	294	478	248	1897	379
TN	11990	12243	12155	12251	9483	58122	11624
Sensitivity	95.1	92.3	93.8	92.7	90.9	92.9	92.9
Precision	16.6	21.4	23.4	21.0	24.4	21.1	21.1
Specificity	96.8	96.2	97.6	96.2	97.5	96.8	96.8
Accuracy	96.8	96.1	97.6	96.2	97.4	96.8	96.8

### Algorithm performance

We evaluated the DeepImpact algorithm with 5-fold cross validation using sensitivity, precision, specificity, and accuracy (Table 3). 95.3% sensitivity and 96.0% precision ensures identification of most header events (low FN), without a high FP rate. Furthermore, we tested the trained DeepImpact algorithm on 5 independent soccer games. These games included 546 header events and 60,018 nonheader events. The DeepImpact algorithm was able to identify 92.9% of header events with only 39 header events missing (Table 4), which corresponds to an average of 8 missing headers per game. With a precision of 21.1%, the algorithm did not perform as well as it did on the validation set, with an average of 379 nonheaders identified as header events in each game.

**Figure 3:**
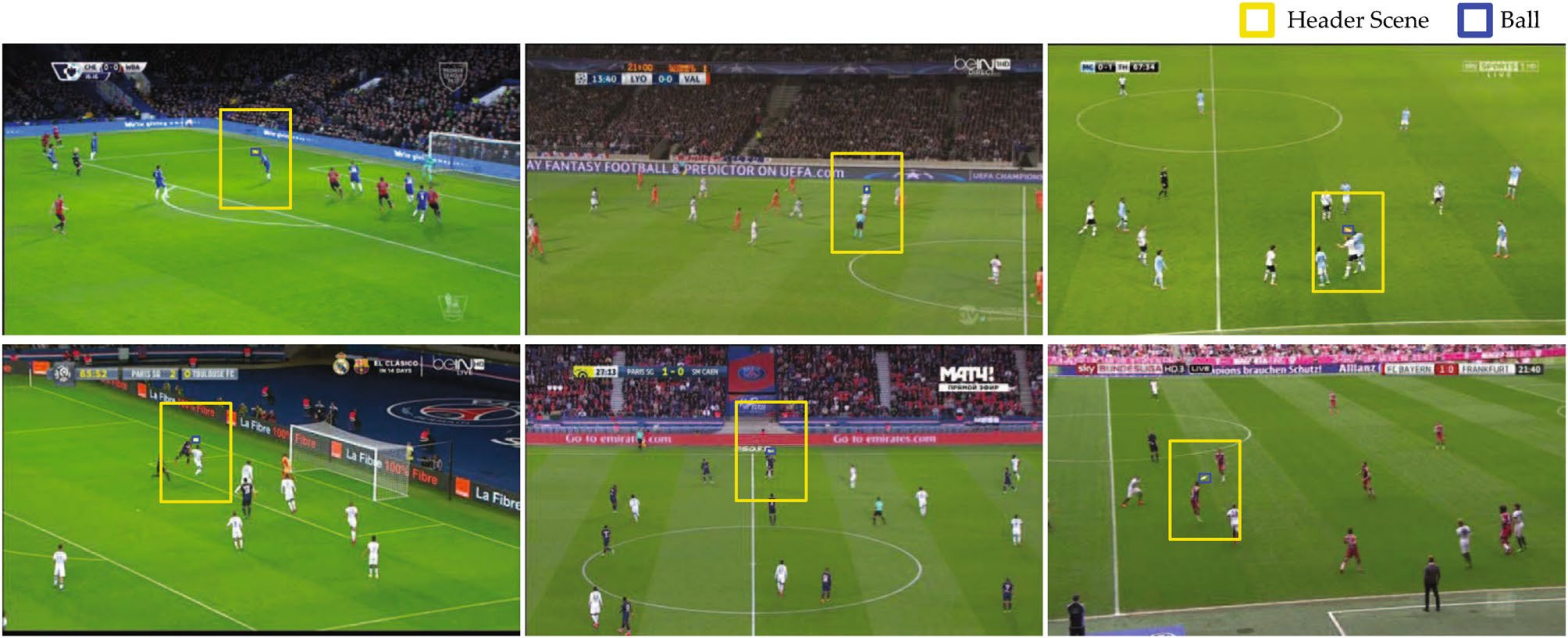
Examples of successful identification of header events. Header events with relatively larger ball size and successful ball detection and tracking were identified correctly.

**Figure 4:**
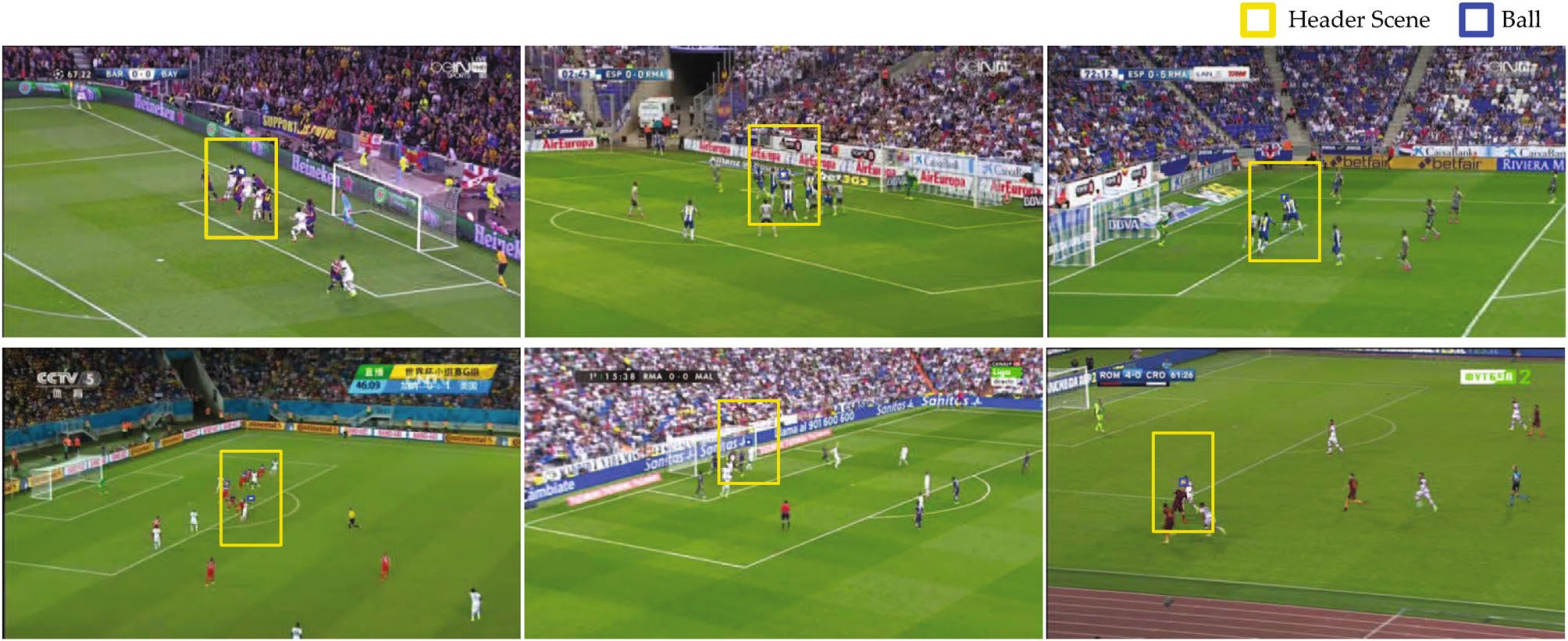
Examples of unsuccessful header detection. Unidentified header events included crowded header scenes (e.g. header after a corner kick) as well as events with the ball being distant from the camera. In these cases, the ball could not be detected in most video frames and thus could not be tracked.

Figures 3 and 4 show samples of successful and unsuccessful identification of header events, respectively. Successful events mostly included events in which the ball was successfully tracked and few players were present in the header scene. Headers that occurred in the centre regions of the soccer field and closer to the camera were more likely to be detected. Unsuccessful events happened when the ball was unidentified in most frames of the video segments. In such cases, the ball could not be tracked and uncropped frames were used by the algorithm. This mostly included crowded events with multiple players in the header’s scene. Headers that happened after a corner kick were the most common case of the failed detections. Other unsuccessful cases included events that were distant from the camera with very small ball size.

We verified that the DeepImpact algorithm indeed requires both clean spatial information (ball tracking and frame cropping using ball position) and temporal information (spatiotemporal feature aggregation) of video segments to accurately identify header events. As shown in Table 5, an image classifier that was trained to classify video frames as header and nonheader without temporal information performed poorly. Adding temporal information improved the performance of the simple classifier on the test dataset by enhancing the sensitivity from 79.1% to 92.1% and the precision from 0.4% to 1.4%. When frames were also cropped using estimated ball position, sensitivity and precision were further improved to 92.9% and 21.1% respectively. Frame cropping contributed to a more substantial improvement in precision.

**Table 5:** Performance of different algorithm designs in header detection.

	Settings		Validation				Test			
	Temporal Information	Frame cropping	SENS	PREC	SPEC	ACU	SENS	PREC	SPEC	ACU
Setting 1			72.5	32.3	77.7	77.1	79.1	0.4	33.9	34.1
Setting 2	$\checkmark$		89.5	90.7	98.7	97.5	92.1	1.4	30.5	31.1
Setting 3	$\checkmark$	$\checkmark$	95.3	96.0	99.4	98.9	92.9	21.1	96.8	96.8

## Discussion

In this work, we present a fully video-based method for detection of soccer header events. Our proposed solution does not require head kinematics data and only uses a single-view video footage of soccer games to identify header events. The DeepImpact algorithm is based on deep learning techniques and uses a data-driven approach to learn the visual and temporal features of header and nonheader events present in the video data. Using this methodology, video-based features can replace usual head kinematics-based features for header detection. To train and validate the algorithm, we also contribute a sizable labeled soccer header video dataset.

### Algorithm performance and application in head impact exposure estimation

In cross validation, our method yielded over 95% performance on all evaluation metrics. In addition, we tested the algorithm in its real use case on five independent full soccer games. In the independent test, 92.9% of the headers were detected with an average of 8 missing events per game. Considering 20 players on the field (excluding goalkeepers), the 8 missing headers correspond to approximately 0.4 missing headers per player per game. In comparison, wearable sensor studies that conducted independent video analysis to verify sensor performance have found lower sensitivity (Table 1).

In the independent full-game test, the precision dropped from the cross validation performance of 96% to 21.1%, with an average of 379 false positive events per game. The difference between validation and test performance can be explained by comparing the validation and test datasets. Header events correspond to a short duration of the total video time of the soccer games, and having a highly imbalanced dataset can complicate network training. We reduced the imbalance in headers/nonheaders during training and validation by including 7 nonheaders for each header event. It is best that both validation and test datasets have similar distribution of classes. In our full-game test dataset, we had an average of 110 nonheader segments for every header segment, i.e. a more imbalanced dataset. Therefore, there was a higher possibility for nonheader events to be identified as headers, leading to relatively higher proportions of false positives. While the precision seems low, it is similar to the performance of some wearable sensors. In fact, sensor overestimation of head impact exposure due to high false positive rate is a common problem, which leads to the best practices approach of further applying video verification on sensor-detected impacts^4,29,31^.

We would like to note that the false positive detection of nonheader video segments could be mitigated by a quick secondary review. Based on the test results, the algorithm could identify around 480 events per game (both TP and FP) as potential header events that require further verification by a video rater. With each event segment consisting of only 11 frames, the total review duration is approximately 3.5 minutes, which is significantly shorter than a full soccer game. As such, although the current network may not yet fully automate accurate header detection, it would streamline and enable efficient video-based measurement of head impact exposure in soccer.

Efficient video-based head impact exposure measurements can contribute datasets substantially larger than currently available sensor-based datasets to study the mechanisms of cumulative brain injury with low resource requirements, especially considering that video recordings are already available for many sports events. Wearable sensors, on the other hand, have limited utility in large-scale exposure studies due to high cost of sensors, substantial logistics burden, user compliance issues, and the necessity of video verification. Therefore, a streamlined process to estimate impact exposure only using video data is a promising solution to address the limitations of wearable sensors. Moreover, video data can further provide information such as impact type and context that are not measurable by impact sensors.

Although video data can be a strong solution to measure impact frequency, an inherent disadvantage of this data is that it cannot be used to obtain accurate impact magnitude information. Both impact frequency and magnitude contribute to our understanding of brain injury. Therefore, combining wearable sensors and our proposed video-based algorithm can provide a promising solution for efficient collection of both impact frequency and magnitude information. While the proposed algorithm can efficiently assess sensor’s sensitivity and collect impact frequency information, wearable sensors can measure the magnitude of the verified impacts.

### Algorithm design

Our framework for video-based header detection utilized two sources of information in video: ball position and temporal information of video frames. Among these elements, accurate ball detection was the most challenging task. In general, ball detection in broadcast soccer videos is not a trivial task^57^. Several factors contribute to the difficulty, including small ball size, variable ball shape due to fast ball/camera movement, occlusion, and ball possession by players for extended durations. While the ball detection network performed well when header events occurred closer to the camera, it generally did not identify the ball in distant events from the camera or when multiple players were in the scene. Since we included nearly 100,000 labeled ball images in the training dataset, containing a diverse set of possible ball locations and sizes, the difficulty in ball detection cannot be fully explained by the size or quality of the training dataset. As shown in Fig. 2, ball position is not easily identifiable even by a human rater in some failure cases. Considering the significant effect of frame cropping in header detection performance (Table 5), improving ball detection using multiple camera views^57^ may help enhance header detection performance. For each video segment, the camera angle with the best performance in detection of ball position can be selected and used for the next steps of the algorithm.

In this work, we used TSM methodology to extract and utilize temporal information of the video segments. In our early trials on smaller versions of the header dataset, we tested long short-term memory (LSTM) networks^58^ and temporal segment networks (TSN)^59^ for temporal feature aggregation. TSM showed the best performance in these early trials. While TSN had a considerably better performance than LSTM in all the metrics, TSM could achieve a slightly better performance than TSN. Thus, we used TSM to conduct our experiments on the full header dataset. Using temporal information for activity recognition in video is an active area of research in computer vision. Future improved temporal feature aggregation techniques may also aid in improving the DeepImpact algorithm.

The dataset used for training DL-based algorithms is an essential element for their successful implementation. The dataset should be large enough and well representative of the real use case of the algorithm. Considering this point, the SoccerNet-v2 and SoccerDB datasets were highly valuable for this work. Using these datasets, we had access to large-scale open-source video datasets, which was crucial to training an effective network in the current project.

### Limitation and future work

In this work, we used single-view broadcast video footage to train our network. This is a limited source of video information. First, 64% of videos in the dataset had low 720p video quality. Second, the broadcast camera angle changes constantly to follow the ball, which can cause blurry ball and players in the images. Third, one video angle provides limited information for distant events happening on the opposite side of the camera location. In addition, impact events may be invisible in the single video view and remain undetected. Fourth, current dataset was selected from videos with different frame per second rates to enlarge the dataset. Having a consistent frame rate could improve the performance of the algorithm. To evaluate the header detection algorithm, we tested the developed network on an independent set of games. We should note that these videos were selected from similar professional-level broadcast soccer games that were used in training the network. Further testing using different video sources would be necessary to evaluate the generalizability of the proposed solution.

The current solution does not identify the player involved in each header. Therefore, in addition to removing false positive detections, further video analysis by raters would be necessary to conduct individual exposure estimates. Furthermore, the DeepImpact algorithm is designed to only detect header events. Other types of impacts such as head to head, head to body, and head to ground will not be identified by the current network. Although the majority of head impacts are header events in soccer, other sports such as American football have different impact scenarios that may require different considerations in network design. In addition, an inherent limitation of video-based impact exposure measurements is the lack of impact kinematics information. It would be substantially more challenging to use video information to estimate the severity of impacts compared to sensor information.

A helpful future step in this research is to add player tracking into the system. Although player tracking using video information alone is a challenging task^60^, its successful implementation has two advantages. First, having players’ position information can complement the DeepImpact algorithm to identify the player in the header event. Second, players’ positions, along with their pose information, may not only provide additional information to confirm headers but also identify other types of impacts such as falling or head-body contacts. Human pose estimation using video has been extensively researched in the computer vision community^61^ and powerful algorithms have been developed for 2-dimensional and 3-dimensional estimation of human pose. Adaption of these algorithms for soccer can be used for impact detection and potentially kinematics estimation.

Future work may also benefit from a multi-view soccer video dataset with better-defined camera position and angle. In fact, with multi-view information, we anticipate that a video-based header detection algorithm would be capable of fully automating accurate identification of header events, as potential false positive and false negative detections may be cross-verified using the multiple views. Another extension of the current work is to adapt the system for head impact detection in other sports such as American football. Head impact events in the video can be characterized differently in each sport. For example, helmet contact with other objects such as other helmets, ground, or another player’s body is the main characteristic of head impacts in American football. These characteristics can be used to modify and adapt the header detection algorithm. Expanding this work to other sports would enable multi-sport head impact exposure measurements, especially for sports where brain injuries are more prevalent.

## Conclusion

We developed DeepImpact, an algorithm to identify header events in soccer using only video data. This algorithm achieved 95.3% sensitivity and 96% precision in cross validation, and 92.9% sensitivity and 21.1% precision on an independent test dataset. This test performance corresponds to correct identification of most header events, with an average of 3.5 minutes of each game video requiring further verification by a video rater to eliminate false positive detections. Our solution is a first step towards video-based head impact exposure quantification in soccer, and can help streamline the process of video analysis for independent or sensor-based impact exposure measurements. Such a system may enable the collection of an accurate large-scale head impact exposure dataset to study potential cumulative brain injury risk, and may also be further developed into an automated exposure monitoring tool for brain injury management in sports.

## Data Availability

The video data that support the findings of this study are available from SoccerDB^41^ and SoccerNet-v2^42^ datasets, but restrictions apply to the availability of these data, which were used under license for the current study, and so are not publicly available. Data are however available from the authors upon reasonable request and with permission from the creators of the original dataset. The label data generated and analysed during the current study are available from the corresponding author on reasonable request.

## Code availability

The code used to generate results of this study is available at https://github.com/AHNR0/DeepImpact.git.
